# MARK1 suppress malignant progression of hepatocellular carcinoma and improves sorafenib resistance through negatively regulating POTEE

**DOI:** 10.1515/med-2024-1060

**Published:** 2024-11-07

**Authors:** Xin Lu, Zhiyuan Chen, Wenting Mi, Jianming Zheng, Yubin Liu

**Affiliations:** Department of Hepatobiliary Surgery, Guangdong Provincial People’s Hospital (Guangdong Academy of Medical Sciences), Southern Medical University, Guangzhou, China; Gastroenterology Department, Nanfang Hospital, Southern Medical University, Guangzhou, China; Department of Hepatobiliary Surgery, Guangdong Provincial People’s Hospital (Guangdong Academy of Medical Sciences), Southern Medical University, 106 Zhongshan Second Road, Yuexiu District, Guangzhou, China

**Keywords:** MARK1, POTEE, hepatocellular carcinoma, sorafenib, malignant progression

## Abstract

**Purpose:**

This study aimed to investigate the role of microtubule-affinity regulatory protein kinase 1 (MARK1) in hepatocellular carcinoma (HCC) progression, its association with sorafenib sensitivity, and the interplay between MARK1 and POTE Ankyrin domain family member E(POTEE) in HCC cells.

**Methods:**

Quantitative real-time polymerase chain reaction analysis was used to assess MARK1 and POTEE expression in 60 pairs of HCC tissues and cell lines. The correlation between MARK1 levels, clinicopathological features, and patient prognosis was analyzed. Sorafenib-resistant HCC cell models were developed, followed by MARK1 overexpression to evaluate its impact on cell functions. Luciferase reporter assays and rescue experiments were conducted to elucidate the MARK1–POTEE regulatory mechanism.

**Results:**

MARK1 exhibited decreased mRNA expression in HCC tissues and cells, correlating with adverse clinicopathological features and poorer patient survival. Luciferase assays confirmed direct binding between MARK1 and POTEE. Sorafenib treatment increased MARK1 protein levels, reduced POTEE, and inhibited cell proliferation. Overexpressing MARK1 suppressed sorafenib-induced proliferation in resistant cells, while co-overexpression of MARK1 and POTEE reversed this effect.

**Conclusion:**

MARK1 potentially restrains HCC progression and enhances sorafenib resistance by negatively modulating POTEE expression, highlighting its significance as a therapeutic target in HCC treatment.

## 1 Introduction

Hepatocellular carcinoma (HCC) is the second leading cause of death induced by cancer in the world, with its characteristics including insidious onset, high recurrence rate, and strong metastasis [1,2]. It is the third largest malignancy in China, which accounts for more than half of the global cases [3,4]. Therefore, it is of great clinical significance to establish a reasonable treatment scheme for HCC and explore new treatment strategies [5,6]. Hepatitis B and C virus infections are leading causes of HCC, particularly in Asia and Africa. Chronic infection with these viruses leads to liver inflammation, cirrhosis, and eventually HCC. Given the high global burden of these infections, it is critical to consider them in HCC pathogenesis [7]. The immune system also plays a critical role in the development and progression of HCC. Pro-inflammatory cytokines, such as TNF-α and IL-6, promote carcinogenesis by activating various signaling pathways. These pathways contribute to tumor cell proliferation, survival, and invasion. Many studies have highlighted the importance of inflammation in cancer development, which we discuss in further detail in this study [8,9]. At present, the precise treatment of tumors, especially molecular targeted drug treatments, has attracted more and more attention. Its unique anti-tumor effect is gradually revealed, and its application in liver cancer has also achieved initial results [10,11]. However, unlike other tumors, there are large heterogeneities in molecular signaling pathways and biological characteristics among different subpopulations of liver cancer, for example, BRAF gene mutations in melanoma account for nearly half, which directly leads to limitation of molecular targeted drugs [12,13].

Existing clinical evidence have showed that sorafenib, as a leader of molecular-targeted drugs, could remarkably extend the survival time of patients with advanced liver cancer and has been first approved by the FDA of USA for the treatment of advanced liver cancer [14,15]. However, due to its limited sensitivity and effectiveness and the great individual differences, its efficacy is far from meeting patients’ expectations [15,16]. Sorafenib is only sensitive to 30% of patients with liver cancer, while the rest 70% patients show different degrees of resistance to sorafenib treatment. Although a number of clinical trials of sorafenib combined therapy have been carried out, no better targeted drug has been found to treat sorafenib-resistant liver cancer [17,18]. Therefore, the identification of biological targets is related to sorafenib sensitivity and individualized treatment of patients with different liver cancer subgroups. On the one hand, it greatly saves social medical resources, and on the other hand, it avoids unnecessary overtreatment of patients [18,19]. In addition, exploring the role of specific molecular targets in the sorafenib sensitivity of HCC cells and the underlying mechanisms can help to provide new ideas for individualized treatment of HCC [19,20].

Microtubule-affinity regulatory protein kinase (MARK), a novel mammalian serine/threonine kinase, is a microtubule-related protein binding domain kinase [21,22]. It is involved in the phosphorylation of tau, MAP2, and MAP4 proteins in microtubule-related fields, leading to microtubule-decomposition and increasing dynamic changes [22]. We explored the role of MARK1 in the malignant progression of HCC and its effect on HCC cell resistance to sorafenib, and further discussed the mutual regulation mechanism between MARK1 and POTEE.

## 2 Patients and methods

### 2.1 Patients and HCC samples

Tumor tissue specimens and adjacent ones of 60 HCC patients undergoing surgical resection were collected. All subjects had not received any radiotherapy or chemotherapy before surgery. HCC classification was based on the 8th edition UICC/AJCC liver cancer TNM staging criteria, which was used to categorize the patients according to tumor size, nodal involvement, and distant metastasis [23].

### 2.2 Cell culture

Human-derived HCC cells (MHCC97H, SMMC-7221, Huh7, Hep3B) and a human normal liver cell line (HL-7702) provided by American Type Culture Collection (Manassas, VA, USA) were cultured with Dulbecco’s Modified Eagle’s Medium (Gibco, Rockville, MD, USA) supplemented with 10% fetal bovine serum (Gibco, Rockville, MD, USA), streptomycin (100 μg/mL), and penicillin (100 U/mL) in an incubator with 5% CO_2_ at 37°C.

### 2.3 Transfection

Lipofectamine 3000 reagent (Invitrogen, Carlsbad, CA, USA) was mixed with MARK1 overexpression sequence (MARK1) (GenePharma, Shanghai, China) and then added into cells when cell density reached 70%. After 48 h, cells were collected for subsequent analysis.

### 2.4 Cell counting kit-8 (CCK-8) assay

After 48 h of transfection, cells were seeded into 60-well plates (2,000 cells/well), and CCK-8 test (Dojindo Molecular Technologies, Kumamoto, Japan) was carried out to detect cell proliferation capacity based on the instructions.

### 2.5 Colony formation experiment

After transfection for 48 h, 200 cells were seeded in each well of a 6-well plate and cultured with complete medium for 2 weeks. After that, the cells were cloned and fixed in 2 mL of methanol for 20 min. After the methanol was aspirated, the cells were stained with 0.1% crystal violet, photographed, and counted under a light-selective environment.

### 2.6 Quantitative real-time polymerase chain reaction (qPCR)

qPCR detection was implemented based on the instructions of SYBR^®^ Premix Ex Taq ™ on StepOne Plus Real-time PCR System (TaKaRa, Tokyo, Japan), with glyceraldehyde 3-phosphate dehydrogenase (GAPDH) as internal reference. Primers used in the qPCR reaction were as follows: MARK1: forward: ′-AGCCAGGCAGTGATTTGAGG-3′, reverse: 5′-CAGTAACGAGGGAGGGCTTC-3′; POTEE: forward: 5′-GTACCACGTCCGTGGAGAAG-3′, reverse: 5′-TGTAGAGCAGTCCTCTTTTGC-3′; GAPDH: forward: 5′-CCTGGCACCCAGCACAAT-3′, reverse: 5′-GCTGATCCACATCTGCTGGAA-3′.

### 2.7 Western blot

Western blot analysis was performed according to standard procedures. The primary antibodies against MARK1, POTEE, and GAPDH, and the secondary antibodies anti-mouse and anti-rabbit were all purchased from Cell Signaling Technology (Danvers, MA, USA). Protein expression levels were quantified using densitometry analysis, and the results were presented as graphs in the corresponding figures. We used ImageJ software to analyze the protein bands, following the protocol outlined in previous work [24].

### 2.8 Dual-luciferase reporter assay

HCC cell lines Huh7 and Hep3B were co-transfected with POTEE/NC and pMIR luciferase reporter plasmids using Lipofectamine 2000. After 48 h of transfection, the dual luciferase reporter assay system (Promega, Madison, WI, USA) was used to normalize the reporter luciferase activity to control firefly luciferase activity.

### 2.9 Statistical analysis

GraphPad Prism 5 V5.01 software (La Jolla, CA, USA) was used for statistical analysis. Differences between two groups were analyzed by using Student’s *t*-test. Comparison between multiple groups was done using one-way ANOVA test followed by *post-hoc* test (least significant difference). Each experiment is repeated at least for three independent experiments, and the data are expressed as *X* ± SD. *P* < 0.05 was considered statistically significant.


**Ethical approval:** This study was approved by the ethics committee of Guangdong Provincial People’s Hospital.
**Informed consent:** Signed written informed consents were obtained from the patients and/or guardians.

## 3 Results

### 3.1 Expression of MARK1 and POTEE in HCC

**Figure 1:**
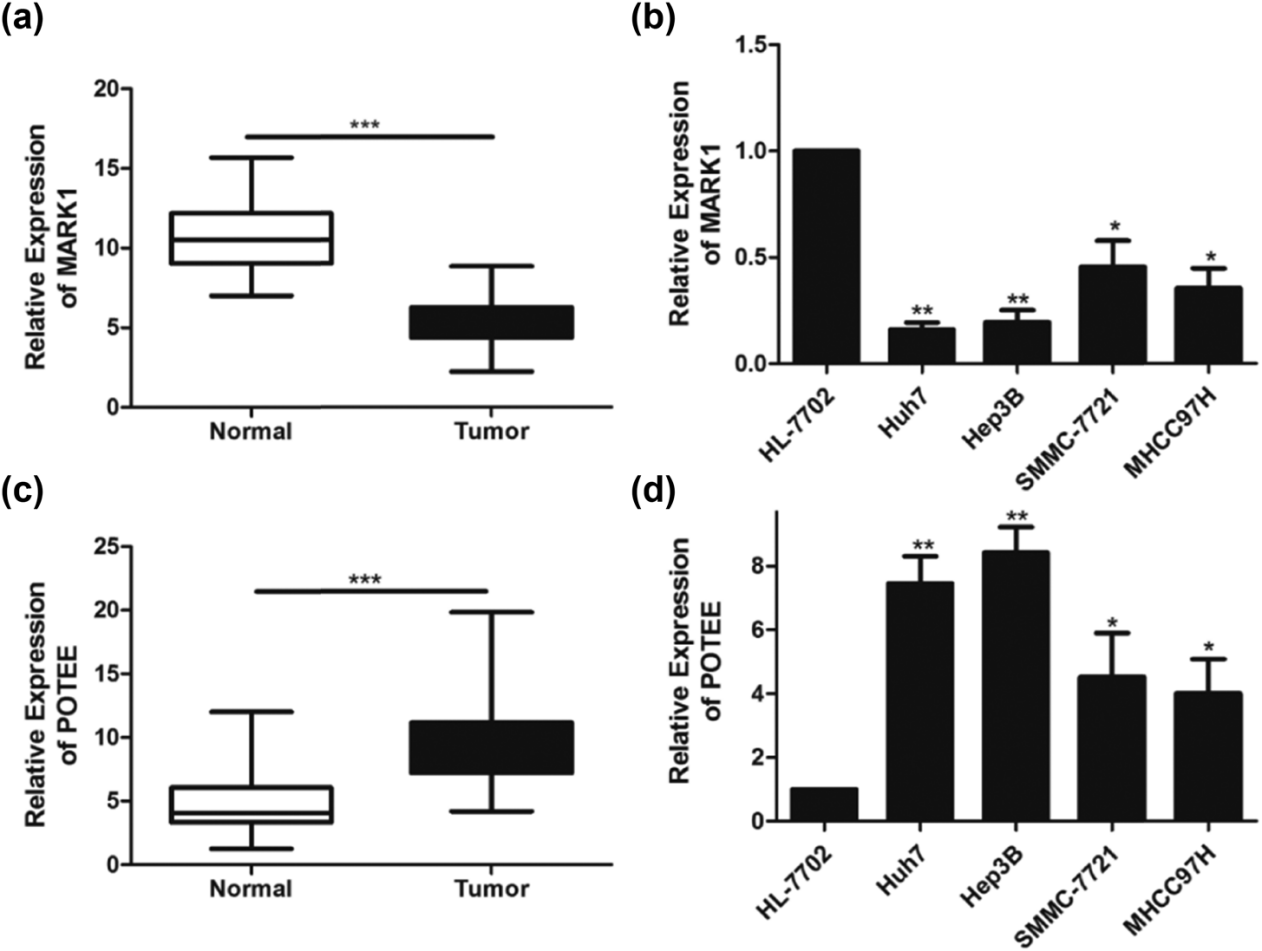
MARK1 and POTEE expression in HCC tissues and cell lines. (a) qRT-PCR was used to detect the difference in the expression of MARK1 in tumor tissues and non-tumor tissues adjacent to the cancer of HCC patients. (b) qRT-PCR was used to detect the expression level of MARK1 in HCC cell lines. (c) qRT-PCR was used to detect the difference in the expression of POTEE in tumor tissues and non-tumor tissues adjacent to cancer of HCC patients. (d) qRT-PCR was used to detect the expression level of POTEE in HCC cell lines. Data are average ± SD, *P < 0.05, **P < 0.01, ***P < 0.001.

To determine the role of MARK1 and POTEE in HCC, a total of 60 pairs of tumor and para-cancer tissue samples from HCC patients were collected and detected their expression in these samples. We found a reduction in MARK1 expression and an increase in POTEE expression in HCC tissues in comparison to the adjacent ones (Figure 1a and c), suggesting that MARK1 may serve as a tumor suppressor gene in this cancer. At the same time, the same alternation in the above two gene levels was observed in HCC cell lines (Figure 1b and d).

### 3.2 MARK1 expression is closely related to the pathological stage of HCC patients

HCC tissues collected from 60 HCC patients were divided into high MARK1 expression and low expression group, and further explored the relationship between MARK1 expression and clinicopathological parameters of HCC patients by chi-square test. Table 1 shows a positive correlation between lowly expressed MARK1 and the pathological stage of HCC, but not with age, gender, incidence of lymph node, or distant metastasis.

**Table 1:** Association of MARK1 expression with clinicopathological characteristics of HCC

Parameters	Number of cases	MARK1 expression		*p*-Value
		High (*n* = 36)	Low (*n* = 24)	
**Age (years)**				0.389
<60	24	16	8	
≥60	36	20	16	
**Gender**				0.058
Male	29	21	8	
Female	31	15	16	
**T stage**				0.008
T1–T2	35	26	9	
T3–T4	25	10	15	
**Lymph node metastasis**				0.914
No	37	22	15	
Yes	23	14	9	
**Distance metastasis**				0.598
No	48	28	20	
Yes	12	8	4	

### 3.3 POTEE is the target gene of MARK1

**Figure 2:**
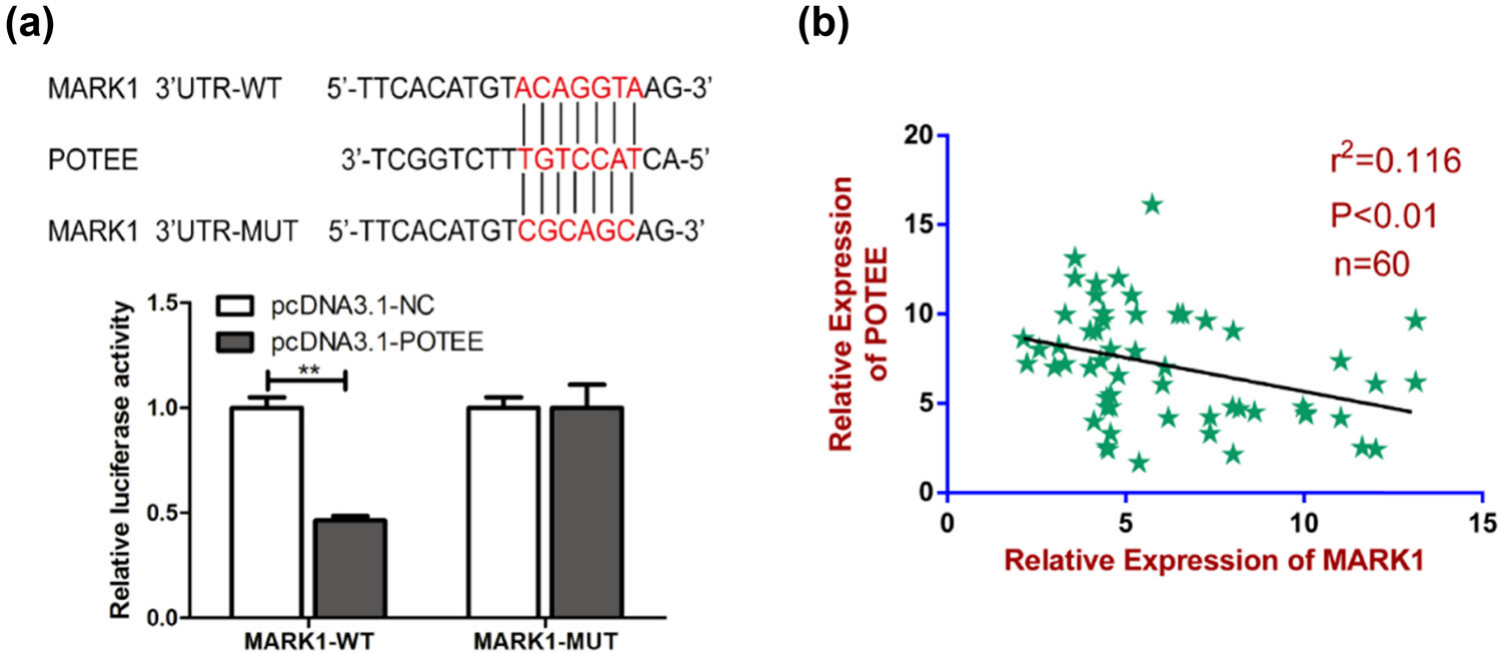
MARK1 directly targets POTEE. (a) The dual luciferase reporter gene experiment verified the direct targeting of MARK1 to POTEE. (b) Expression levels of MARK1 and POTEE in HCC tissues were significantly negatively correlated. Data are average ± SD, **P < 0.01.

Bioinformatics analysis suggested that POTEE is a potential target gene of MARK1, which was further verified by the results obtained from luciferase reporter gene experiments (Figure 2a). In addition, we demonstrated a negative correlation between the mRNA level of MARK1 and POTEE (Figure 2b).

### 3.4 Sorafenib inhibits the growth of HCC cells

**Figure 3:**
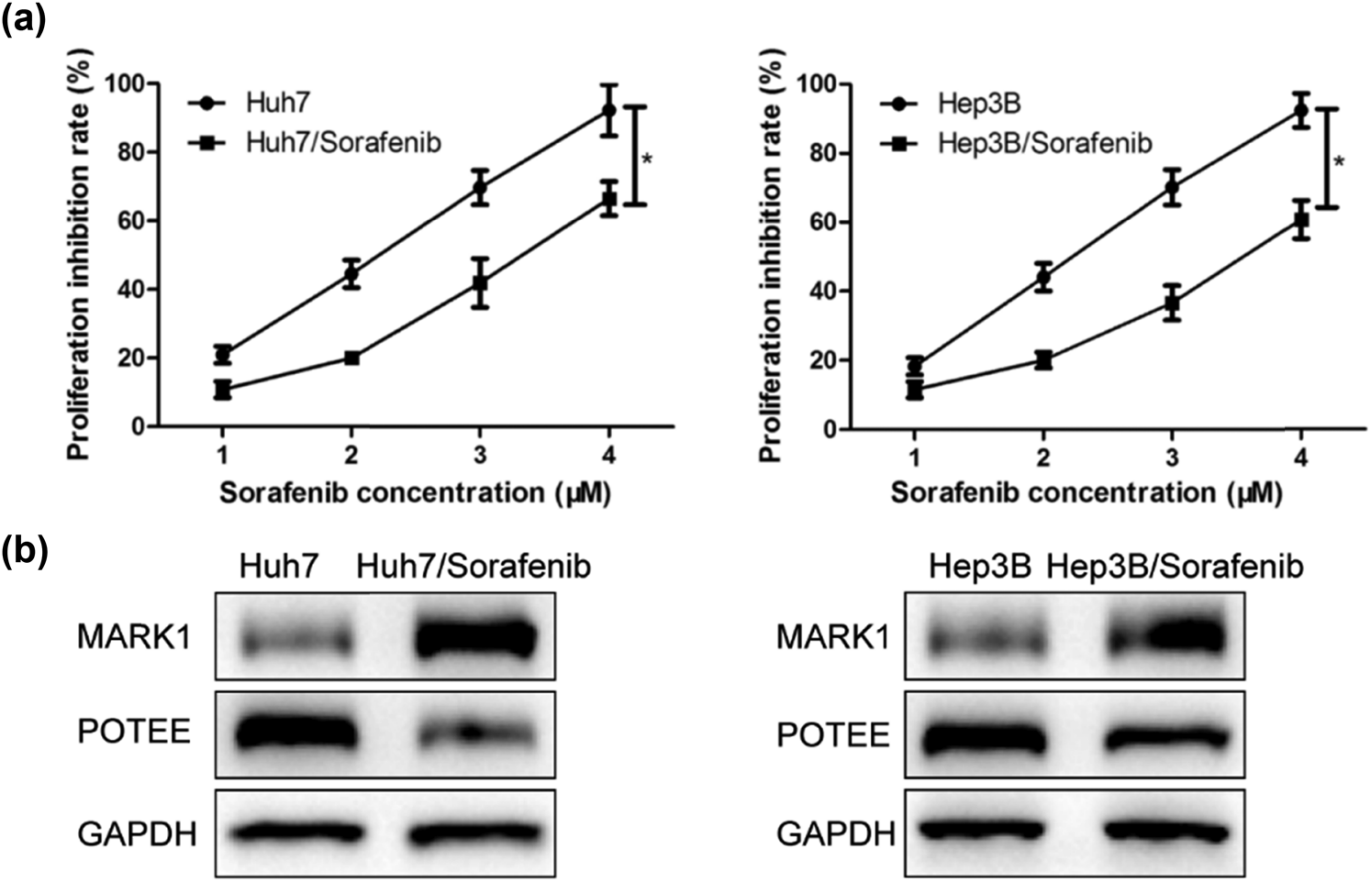
MARK1 can increase the sorafenib sensitivity of HCC cell lines. (a) CCK-8 was used to evaluate the cell proliferation of HCC cell lines Huh7 and Hep3B after treatment of different concentrations of sorafenib. (b) Western blot detected the expression levels of MARK1 and POTEE in normal HCC cells and sorafenib-resistant HCC cells. Data are average ± SD, *P < 0.05.

We then examined the sorafenib sensitivity of HCC cells in Huh7 and Hep3B cell lines by CCK-8 test. As a result, sorafenib dose-dependently suppressed the proliferative ability of HCC cells (Figure 3a). In addition, western blot showed that MARK1 protein level was significantly increased while that of POTEE protein was oppositely reduced in sorafenib-resistant HCC cells compared to normal HCC cells (Figure 3b).

### 3.5 Overexpression of MARK1 suppresses the proliferation of sorafenib-resistant HCC cells

**Figure 4:**
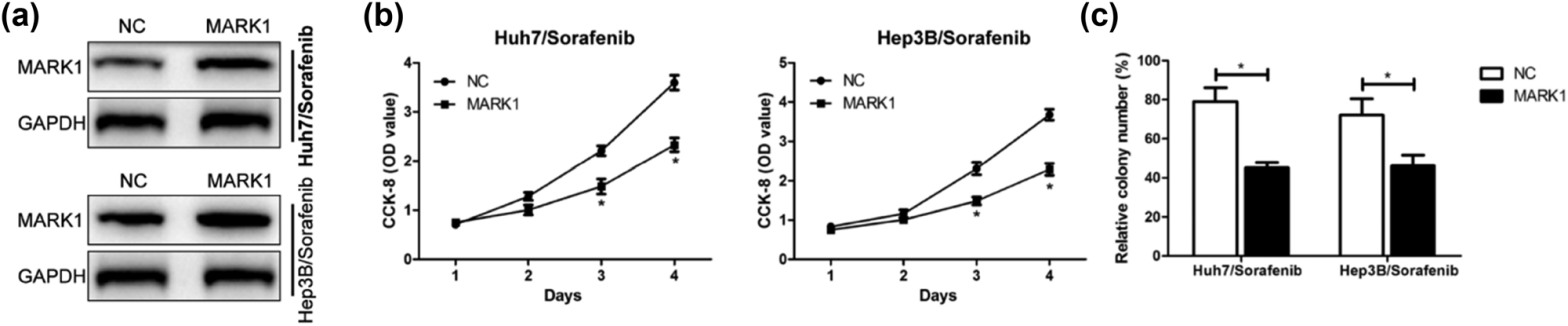
Proliferation of sorafenib-resistant liver cancer cells was inhibited after overexpression of MARK1. (a) Western blot verified the transfection efficiency of sorafenib-resistant HCC cell lines Huh7 and Hep3B after transfection of MARK1 overexpression vector. (b) CCK-8 test was used to detect the cell proliferation ability after transfection of MARK1 overexpression vector in sorafenib-resistant HCC cell lines Huh7 and Hep3B. (c) Plate cloning experiment detected the number of HCC positive proliferating cells (magnification: 40×) after transfection of MARK1 overexpression vector in sorafenib-resistant HCC cell lines Huh7 and Hep3B. Data are average ± SD, *P < 0.05.

Administration of vectors to overexpress MARK1 successfully increases the protein level of MARK1 in sorafenib-resistant cell lines (Figure 4a). Figure 4b shows that overexpressing MARK1 markedly attenuated the proliferation ability of the sorafenib-resistant cells. And the plate cloning experiment also revealed a consistent reduced cell proliferation to CCK8 results (Figure 4c).

### 3.6 POTEE can reverse influence of MARK1 on the proliferative ability of sorafenib-resistant HCC cells

**Figure 5:**
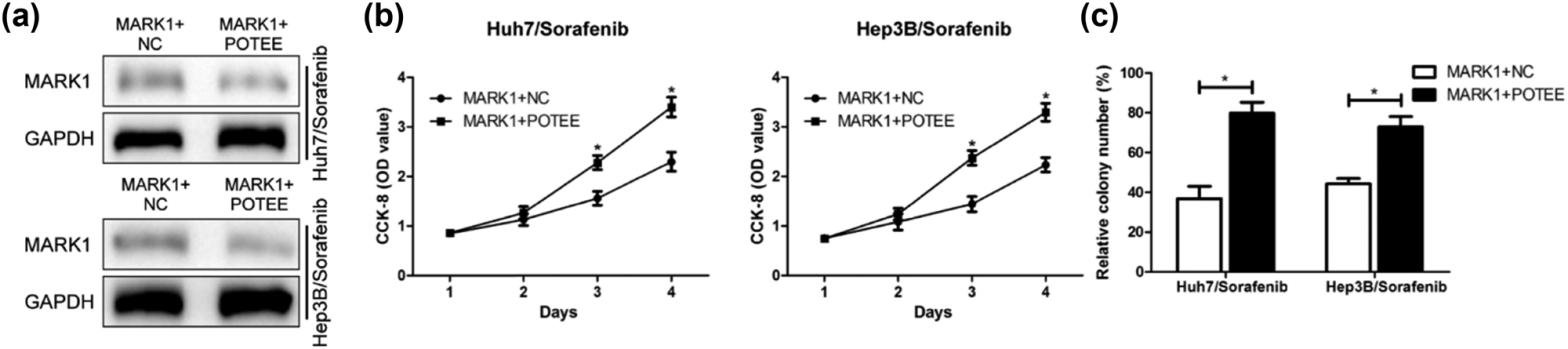
MARK1 can regulate the expression of POTEE in sorafenib-resistant HCC cells. (a) Western blot detected the expression of MARK1 after co-transfection of MARK1 and POTEE overexpression vectors in sorafenib-resistant HCC cell lines Huh7 and Hep3B. (b) CCK-8 experiment was used to detect the effect of co-transfection of MARK1 and POTEE overexpression vectors on proliferation of sorafenib-resistant HCC cells. (c) Plate cloning experiment detected the number of HCC positive proliferating cells (magnification: 40×) after co-transfection of MARK1 and POTEE overexpression vectors in sorafenib-resistant HCC cell lines Huh7 and Hep3B. Data are average ± SD, *P < 0.05.

To further test the interaction between MARK1 and POTEE in sorafenib-resistant cell lines, we used a combination of plasmids to overexpress MARK1 and POTEE *in vitro*. Western blot results confirmed that MARK1 level was remarkably reduced after co-transfection of MARK1 and POTEE overexpression vectors (Figure 5a). Subsequently, both the CCK-8 and the plate cloning experiments showed that overexpression of POTEE could counteract the inhibitory impact of overexpression of MARK1 on sorafenib-resistant HCC cell proliferation capacity (Figure 5b and c).

## 4 Discussion

HCC is one of the most common malignancies in China and even the world, ranking fifth in the incidence of global tumors and third in mortality [1,2,3]. Although great progress has been made in the treatment of HCC with chemotherapy drugs in recent years, the clinical application of chemotherapy drugs has been seriously hindered due to the huge difference in efficacy among different HCC patients, which has become a major obstacle to the treatment of HCC [4,5,6]. Sorafenib, an oral multi-kinase inhibitor, is a targeted drug used in the clinical treatment of advanced HCC in recent years. Tumor angiogenesis is blocked by inhibiting vascular endothelial growth factor receptor and platelet-derived growth factor receptor [15,16,17,18]. Although sorafenib is able to remarkably extend the survival time of some HCC patients, with the emergence of primary and acquired resistance to sorafenib, the exploration of biological targets related to sorafenib sensitivity in HCC becomes increasingly important [18,19,20].

In this study, a large number of clinical samples of HCC patients for the first time were used to investigate the role of MARK1 and POTEE in the development of this cancer. qPCR results showed that MARK1 expression in HCC cancer tissues and cell lines was decreased compared to that in para-carcinoma tissues and normal liver cell lines, while POTEE was increased to varying degrees, indicating that MARK1 and POTEE play an essential part in the progression of HCC. To further verify the relationship between MARK1 and sorafenib sensitivity of HCC cells, we conducted CCK-8 test, which then demonstrated that sorafenib suppresses the proliferative capacity. Meanwhile, MARK1 level in HCC cells with sorafenib resistance was remarkably increased, while POTEE level was decreased. To verify the influence of MARK1 on the biological behavior of sorafenib-resistant HCC cell lines, we overexpressed MARK1 and measured the proliferation of these cells. As a result, we found that MARK1 is able to inhibit the proliferation capacity of HCC and plays a pivotal role in HCC, but the specific molecular mechanism remains unclear. Luciferase reporter assay verified that MARK1 can directly bind to its target gene POTEE, and we demonstrated that overexpression of POTEE could reverse the suppressing influence of overexpression of MARK on the proliferative ability of sorafenib-resistant HCC cells. Therefore, this study suggests that MARK1 may inhibit the malignant progression of HCC and promote sorafenib resistance through regulating POTEE.

This study is just a preliminary study on part of the mechanism of MARK1’s negative regulation of POTEE on liver cancer cells. The specific mechanism is much more complex than we imagined, and further research is needed to provide new ideas for the treatment of liver cancer.

## 5 Conclusions

This study suggests that MARK1 may inhibit malignant progression of HCC and increase HCC cell resistance to sorafenib through negatively modulating POTEE expression.
